# Reevaluating Rifampicin Breakpoint Concentrations for Mycobacterium tuberculosis Isolates with Disputed *rpoB* Mutations and Discordant Susceptibility Phenotypes

**DOI:** 10.1128/spectrum.02087-21

**Published:** 2022-02-02

**Authors:** Wei Wang, Rongmei Liu, Cong Yao, Fengmin Huo, Yuanyuan Shang, Xuxia Zhang, Yufeng Wang, Zhongtan Xue, Liping Ma, Yu Pang

**Affiliations:** a Department of Bacteriology and Immunology, Beijing Key Laboratory on Drug-Resistant Tuberculosis Research, Beijing Tuberculosis and Thoracic Tumor Research Institute/Beijing Chest Hospital, Capital Medical University, Beijing, People’s Republic of China; b Department of Tuberculosis, Beijing Tuberculosis and Thoracic Tumor Research Institute/Beijing Chest Hospital, Capital Medical University, Beijing, People’s Republic of China; c National Tuberculosis Clinical Laboratory, Beijing Tuberculosis and Thoracic Tumor Research Institute/Beijing Chest Hospital, Capital Medical University, Beijing, People’s Republic of China; d Innovation Alliance on Tuberculosis Diagnosis and Treatment, Beijing, People’s Republic of China; Keck School of Medicine of the University of Southern California

**Keywords:** mutations, *Mycobacterium tuberculosis*, rifampicin, susceptibility, rpoB

## Abstract

In this study, rifampicin resistance breakpoints based on MICs of disrupted *rpoB* mutants of Mycobacterium tuberculosis (MTB) were explored using the Mycobacteria Growth Indicator Tube (MGIT) system and microplate alamarBlue assay (MABA). Sixty-one MTB isolates with disputed low-level rifampicin resistance-associated *rpoB* mutations and 40 RIF-susceptible wild-type isolates were included. Among the 61 resistant isolates, 25 (41.0%) had MICs ≥2.0 mg/L via MABA, while 16 (26.2%) were identified as RIF resistant via MGIT. Epidemiological cut-off (ECOFF) values obtained using MABA and MGIT were 0.25 and 0.125 mg/L, respectively. Based on 0.125 mg/L as a tentative critical concentration (CC), MABA RIF resistance-detection sensitivity was 93.4%, prompting the reduction of the MGIT CC to 0.125 mg/L, given that only a single isolate (1.6%) with the borderline mutation would be misclassified as susceptible to RIF based on this CC. Based on DNA sequencing of RRDR as the gold standard, the diagnostic accuracy of MGIT (99.0%) was significantly higher than that of MABA (91.1%). MICs of Leu^511^Pro mutant isolates were negatively correlated with time to liquid culture positivity (TTP) in our analysis (*R* = 0.957, *P* < 0.01). In conclusion, our results demonstrated missed detection of a high proportion of rifampicin-resistant isolates based on the WHO-endorsed CC. Such missed detections would be avoided by reducing the optimal MGIT RIF CC to 0.125 mg/L. In addition, MGIT based on reduced CC outperformed MABA in detecting borderline RIF resistance, with MABA MIC results obtained for isolates with the same mutation correlating with MTB growth rate.

**IMPORTANCE** Tuberculosis (TB) is still one of the world's leading infectious disease killers. The early and accurate diagnosis of RIF resistance is necessary to deliver timely and appropriate treatment for TB patients and improve their clinical outcome. Actually, a proportion of MTB isolates with disputed rpoB mutations present a diagnostic dilemma between Xpert and phenotypical drug susceptibility testing (pDST). Recently, WHO reported a pragmatic approach by lowering critical concentration (CC) to boost sensitivity of resistance detection of pDST. Therefore, a detailed analysis of the association between RIF susceptibility and disrupted mutations within rpoB gene would lay a foundation to assess the diagnostic accuracy of pDST with lowering RIF CC. In this study, we aim to determine the MICs of MTB isolates with disrupted mutations by MGIT and microplate alamarBlue assay (MABA). We also aimed to determine the optimal breakpoints for MTB isolates with these mutations.

## INTRODUCTION

Despite great progress in tuberculosis (TB) control, approximately 10.0 million incident TB cases and 1.5 million deaths globally were reported in 2021 (1). The emergence of drug-resistant TB, especially multidrug-resistant TB defined as resistance to both rifampicin (RIF) and isoniazid (INH), has further fueled the epidemic of this infectious disease (2). RIF plays a pivotal role in TB treatment, due to its potent bactericidal effects (3, 4), but RIF resistance has emerged that has prompted the World Health Organization (WHO) to recommend that RIF-resistant TB (RR-TB) patients receive second-line therapy (5). Thus, early and accurate diagnosis of RR-TB is necessary to enable timely administration of appropriate treatments to improve clinical outcomes.

WHO has endorsed multiple phenotypic methods for determining *in vitro* drug susceptibilities of Mycobacterium tuberculosis (MTB) isolates, including the solid medium-based agar proportion method and the Mycobacteria Growth Indicator Tube (MGIT)-based method utilizing the Bactec MGIT 960 automated system. By continuously monitoring fluorescence levels associated with growing mycobacteria, the MGIT method saves time relative to other drug susceptibility testing methods by providing results in 10 to 12 days (6, 7). Another method, the microplate alamarBlue assay (MABA), has advantages as a broth-based method, due to its dependence on liquid medium supporting rapid MTB growth, thus shortening phenotypic drug susceptibility testing (pDST) turnaround time. However, MABA lacks a WHO endorsement for use in determining MICs of MTB isolates and requires at least 7 days to yield results (8). Currently, molecular diagnostics-based methods are in widespread use that can achieve earlier MTB detection to ensure early TB diagnosis and initiation of effective treatments tailored to individual patients (9). One such assay, GeneXpert MTB/RIF (Xpert, Cephid, USA), is a commercial automated nucleic amplification assay that has been endorsed by WHO for detection of tubercle bacilli as well as RIF resistance (10). In fact, Xpert results are available within 2 h, as compared with multiple months of time required for pDST to yield results. Nevertheless, despite high agreement of results obtained using conventional DST method and GeneXpert MTB/RIF, a small proportion of RIF-resistant MTB isolates with disputed *rpoB* mutations are actually scored as susceptible to RIF, creating a diagnostic dilemma (11, 12). Recently, an official WHO report offered a pragmatic approach to solving this problem by recommending that the critical concentration (CC) be reduced on order to boost sensitivity of pDST resistance detection (13). This recommendation prompted us to conduct a detailed analysis of the association between RIF susceptibility and disputed *rpoB* gene mutations in order to assess diagnostic accuracy of pDST results based on a reduced RIF CC. Although several previous reports had investigated the distribution of MICs of MTB isolates with disputed *rpoB* mutations (11, 14), most studies had limitations stemming from small sample sizes due to low prevalence rates of these mutants.

To avoid such limitations, we carried out an experimental study of MICs determined using MGIT and MABA of MTB isolates with disputed *rpoB* mutations then determined optimal RIF-resistance breakpoints for use in correctly interpreting MIC results for these isolates.

## RESULTS

### MIC results as determined using MGIT and MABA.

**FIG 1:**
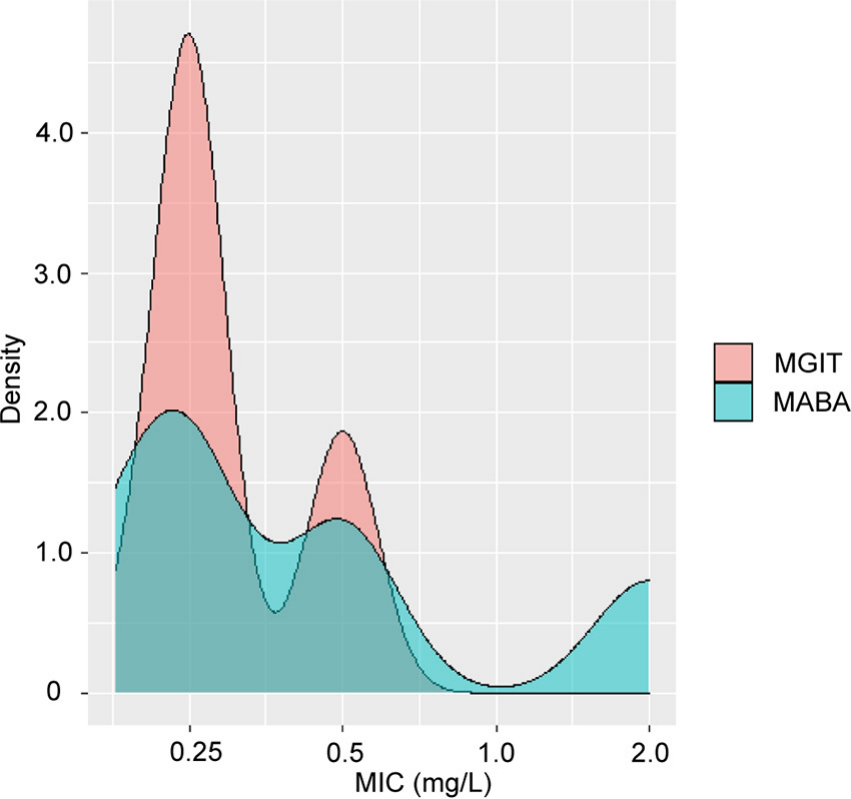
Kernel density estimations of MIC values as determined using MGIT and MABA methods.

We first analyzed the distribution of MICs among isolates with disputed *rpoB* mutations using MGIT and MABA. As summarized in Table 1, of 61 mutant isolates tested via MABA, 25 (40.1%) had MICs of ≥2.0 mg/L that ranged from 2.0 mg/L to 16.0 mg/L, while MGIT identified 16 (26.2%) isolates as RIF resistant. Of note, the distribution of MICs obtained using MABA was more diverse than that obtained using MGIT. As illustrated in Fig. 1, results of kernel density estimations revealed that MICs determined via MGIT of isolates with the Leu^511^Pro mutation were concentrated within the range of 0.25 to 0.5 mg/L, whereas MIC values determined via MABA for these isolates fell within a broader concentration distribution range, from 0.25 to 2.0 mg/L. Meanwhile, MICs of pan-susceptible MTB ranged from 0.031 to 0.25 mg/L and MGTI MICs ranged from 0.031 to 0.13 mg/L; the distribution of MGIT MICs was slightly skewed to higher values as compared with that of MABA MICs, although this difference was not statistically significant.

**TABLE 1:** Distribution of MTB isolates with different MICs stratified by *rpoB* genotypes^*a*^

Method	Mutation type	No. of isolates with different MICs (mg/L)										Total
		0.031	0.063	0.13	0.25	0.5	1.0	2.0	4.0	8.0	16	
MABA	Leu^511^Pro	0	0	4	8	6	4	0	0	0	0	22
	Asp^516^Tyr	0	0	0	2	1	4	0	0	0	0	7
	His^526^Asn	0	0	0	1	1	1	1	0	0	0	4
	His^526^Leu	0	0	0	0	0	0	2	6	2	0	10
	Leu^533^Pro	0	0	0	0	0	4	8	3	3	0	18
	Wild-type	4	23	8	5	0	0	0	0	0	0	40
MGIT	Leu^511^Pro	0	0	1	15	6	0	0	0	0	0	22
	Asp^516^Tyr	0	0	0	2	5	0	0	0	0	0	7
	His^526^Asn	0	0	0	0	2	2	0	0	0	0	4
	His^526^Leu	0	0	0	0	0	0	4	6	0	0	10
	Leu^533^Pro	0	0	0	0	0	12	6	0	0	0	18
	Wild-type	24	11	5	0	0	0	0	0	0	0	40

a MABA, microplate alamarBlue assay; MGIT, Mycobacteria growth indicator tube.

### Effects of reducing CC on diagnostic accuracy.

ECOFF values obtained for MABA and MGIT results were 0.25 and 0.125 mg/L, respectively, which were lower than the current WHO-endorsed CC breakpoint of 0.5 mg/L. This result prompted us to model the effects of different CCs on diagnostic accuracy of MABA and MGIT detection of phenotypic resistance. As summarized in Table 2, sensitivities of the two pDST methods were dramatically increased by reducing the CCs. For MABA implemented based on CC that was tentatively set to 0.125 mg/L, 57 of 61 MTB isolates with disputed mutations were correctly identified as RIF resistant using this CC, yielding a sensitivity of 93.4% for detection of RIF resistance (95% CI = 87.2, 99.7). However, five of 40 RIF-susceptible isolates were misclassified as RIF-resistant at this concentration, for a specificity of 87.5% (95% CI = 77.3, 97.7). Based on these results, the MGIT CC breakpoint was reduced to 0.125 mg/L, given that only a single isolate (1.6%) with a borderline mutation would be misclassified as RIF susceptible when using this CC. In addition, all RIF-susceptible isolates were correctly detected via MGIT, demonstrating a specificity of 100.0% (95% CI = 100.0, 100.0). MGIT, considered the gold standard method, is based on DNA sequencing of the rifampicin resistance-determining region (RRDR) and in this study had a diagnostic accuracy rate of 99.0% (95% CI = 97.1, 100.0), a rate that was significantly higher than the corresponding rate for MABA of 91.1% (95% CI = 85.5, 96.6).

**TABLE 2:** Effect of changing the critical concentration for RIF DST on MGIT or MABA for detection of MTB isolates with disrupted *rpoB* mutations^*a*^

Critical concn	MABA			MGIT		
	Sensitivity % (95% CI)	Specificity % (95% CI)	Accuracy % (95% CI)	Sensitivity % (95% CI)	Specificity % (95% CI)	Accuracy % (95% CI)
1.0 mg/L	41.0 (28.6, 53.3)	100 (100.0, 100.0)	64.4 (55.0, 73.7)	26.2 (15.2, 37.3)	100 (100.0, 100.0)	55.4 (45.8, 65.1)
0.5 mg/L	62.3 (50.1, 74.5)	100 (100.0, 100.0)	77.2 (69.0, 85.4)	49.2 (36.6, 61.7)	100 (100.0, 100.0)	75.2 (66.8, 83.7)
0.25 mg/L	75.4 (64.6, 86.2)	100 (100.0, 100.0)	85.1 (78.2, 92.1)	70.5 (59.0, 81.9)	100 (100.0, 100.0)	82.2 (74.7, 89.6)
0.125 mg/L	93.4 (87.2, 99.7)	87.5 (77.3, 97.7)	91.1 (85.5, 96.6)	98.4 (95.2, 100.0)	100 (100.0, 100.0)	99.0 (97.1, 100.0)

a MABA, microplate alamarBlue assay; MGIT, Mycobacteria growth indicator tube.

### Correlations between MABA MICs and time to liquid culture positivity.

**FIG 2:**
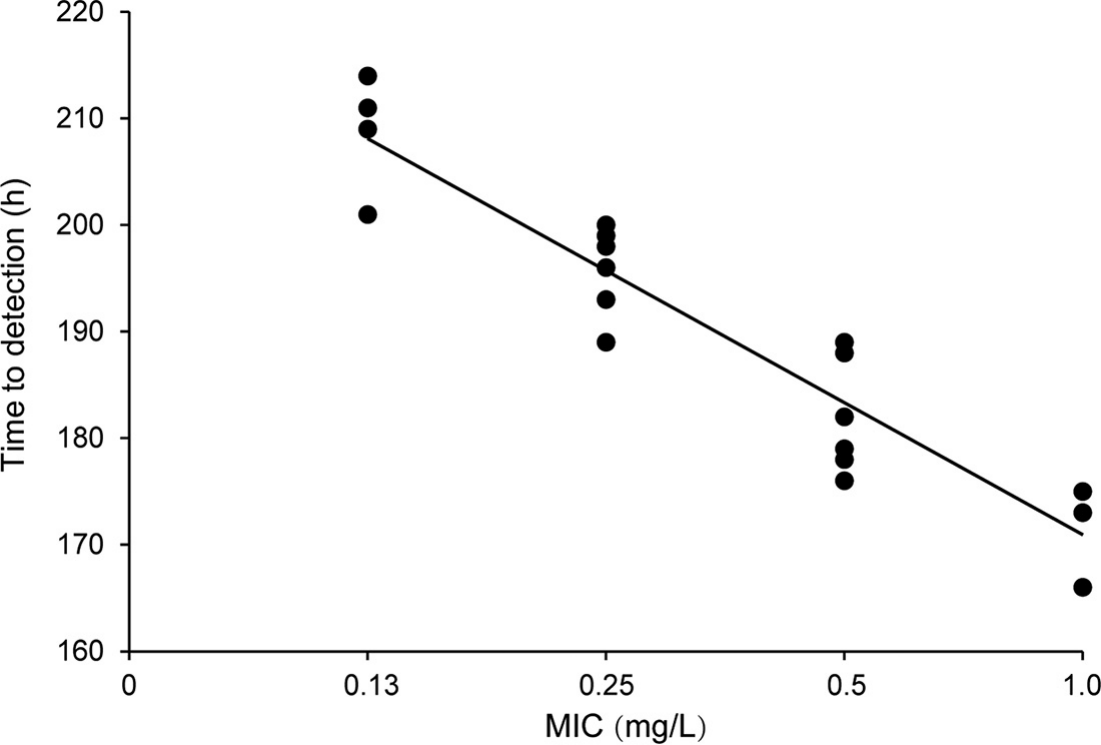
Correlation between MIC values and time to detection of growth of MTB isolates with Leu^511^Pro mutations.

As mentioned above, MGIT diagnostic ability for identifying RIF resistance associated with disrupted mutations was greater than that of MABA, a result that was largely due to a high level of variability of MABA MICs associated with isolates with disputed mutations. We next explored the correlation between MICs obtained using MABA and time to liquid culture positivity (TTP) results then conducted MGIT to analyze TTPs of MTB isolates with the *rpoB* mutation encoding the Leu^511^Pro substitution. As shown in Fig. 2, the average TTP of Leu^511^Pro mutants was 172 h for the 1.0 mg/L group, a value that was significantly lower than values of 182 h obtained for the 0.5 mg/L group, 196 h for 0.25 mg/L group, and 209 h for the 0.125 mg/L group. Ultimately, these results indicated that MICs of MTB isolates were negatively correlated with TTP values in our analysis (*R* = 0.957, *P* < 0.01).

## DISCUSSION

Accurate diagnosis of RIF-resistant TB and timely initiation of optimal treatment are crucial for the success of TB control efforts (15). Despite its reputation as the gold standard of detection of *in vitro* susceptibility, increasing evidence has demonstrated that pDST probably fails to detect MTB isolates with low-level resistance associated with disputed mutations (11, 16). In this study, we investigated MIC distributions of isolates with borderline resistance via MGIT and MABA. Our results demonstrated that a high proportion of these isolates would not be detected based on a CC of 0.5 mg/L, especially isolates with Leu^511^Pro and Asp^516^Tyr substitutions. It is worth noting that after this CC breakpoint was reduced to 0.125 mg/L, only one isolate would be misclassified as RIF susceptible without yielding false positive RIF resistance results for wild-type isolates. Thus, based on these results we recommend that the MGIT RIF CC breakpoint should be lowered to 0.125 mg/L in order to significantly boost the sensitivity of pDST detection of RIF resistance.

We also observed that MGIT based on reduced CC outperformed MABA in detecting borderline RIF resistance, with sensitivity discrepancies largely due to variations in MIC values among isolates possessing the same RRDR point mutation. Although MABA and MGIT are both used for MIC determinations, MGIT is a modified Middlebrook 7H9 broth-based proportion method (17), while MABA is a microdilution assay based on direct observation of bacterial growth (18). Therefore, several factors unique to the MABA method may dramatically affect MABA MIC results without affecting MGIT results, including inoculum size and bacterial growth rate. In an experimental study by Banfi and colleagues, a change in culture inoculum size led to significantly altered streptomycin MICs (18), highlighting the importance of using a standardized inoculum size in order to correctly determine MABA MICs. However, the scaling up of the MABA method would pose a great challenge to clinical laboratories in resource-limited settings.

It is noteworthy that variability in isolate growth rates may explain why isolates with the same mutation exhibited different MICs. Although the fitness cost of a given mutation has not previously been compared among different MTB isolates (19), our experimental data first demonstrated that MICs of MTB isolates with the same mutation correlated with MTB growth rate. Growth rate is a useful indicator for assessing an organism’s fitness costs associated with genetic loss-of-function mutations (20). Conventionally, MTB isolates with a given mutation had comparable fitness costs regardless of their genetic backgrounds. In fact, several factors appeared to influence fitness costs of MTB isolates. On the one hand, variations in *rpoA* or *rpoC* gene functions could, at least in part, compensate for fitness costs of RIF resistance due to *rpoB* mutations (21). Thus, the co-occurrence of compensatory mutations in RIF-resistant isolates could significantly improve the bacterial *in vitro* growth rate. On the other hand, besides mutations within *rpoB*, MTB isolates always harbor multiple additional mutations conferring drug resistance that themselves incur uncertain fitness costs. Despite difficulties associated with accurately estimating fitness costs, diverse genetic mutations undoubtedly lead to diverse impacts on fitness. Thus, it may not be possible to obtain precise MIC determinations for MTB isolates with variable growth rates.

Taken together, our findings have several important implications for the clinical management of RIF-resistant TB patients. First, isolates with borderline RRDR mutations may be misclassified as RIF susceptible, thereby leading to inclusion of RIF in treatment regimens even though RIF would be ineffective, with negative impacts on treatment outcomes. Recently, WHO has declared that any mutations within the RRDR of the *rpoB* gene (except for silent mutations) are assumed to be associated with RIF resistance (22). Consequently, clinicians should be aware that RIF-resistant cases, as determined using Xpert, and RIF susceptible cases, as determined using pDST methods, may be associated with disputed RRDR mutations. Second, *in vitro* studies on MTB RIF and rifabutin cross-resistance have demonstrated that isolates with disputed mutations did not exhibit rifabutin resistance (23, 24) and thus could be effectively treated with rifabutin so that new antibiotic agents could be reserved for difficult cases that cannot be treated with the current antibiotic arsenal. Nevertheless, there is a critical need to retrospectively investigate the clinical response of rifabutin-treated patients harboring disputed mutations in order to confirm treatment efficacy of this antibiotic in such cases.

In conclusion, our results demonstrate that a high proportion of MTB RIF-resistant isolates would not be detected based on WHO-endorsed CC breakpoints, especially for isolates with Leu^511^Pro and Asp^516^Tyr substitutions. Thus, the optimal MGIT RIF CC should be reduced to 0.125 mg/L in order to significantly boost the sensitivity of pDST for detecting RIF resistance. In addition, MGIT based on reduced CC outperformed MABA in detecting borderline RIF resistance. MABA MICs of MTB isolates possessing the same *rpoB* mutation correlated with growth rates of tubercle bacilli. Based on our findings, in order to conduct precise MIC determinations for MTB isolates with variable growth rates, pDST methods should be validated in the future.

## MATERIALS AND METHODS

### Bacterial isolates and culture conditions.

Sixty-one MTB isolates with disputed *rpoB* mutations, defined as mutations conferring MIC values close to the rifampicin breakpoint, were obtained from the Biobank of Tuberculosis of Beijing Chest Hospital. These isolates with *rpoB* mutations encoding RNA polymerase β subunit amino acid substitutions that included 22 with Leu^511^Pro, 7 with Asp^516^Tyr, four with His^526^Asn, 10 with His^526^Leu, and 18 with Leu^533^Pro, as previously described (11). To assess the specificity of various breakpoints for discriminating between resistant and susceptible MTB isolates, the other 40 RIF-susceptible wild-type isolates were evaluated using analysis of MICs obtained via conventional phenotypic DST, as previously described (11). In addition, *rpoB* sequences of MTB isolates were analyzed via Sanger sequencing (20). All bacterial cells were stored at −80°C in Middlebrook 7H9 medium with 10% oleic acid-albumin-dextrose-catalase complex (OADC) (Becton Dickinson, MD, USA) and 5% glycerol. Prior to *in vitro* analysis, isolates were subcultured on Löwenstein-Jensen medium for 4 weeks at 37°C. All experiments were performed under enhanced biosafety level-2 (BSL-2) conditions with appropriate laboratory equipment according to national guidelines.

### MIC determinations.

MICs of MTB isolates were determined via MGIT and microdilution broth-based methods. Mycobacterial suspensions were prepared from 4-week-old colonies on L-J medium. Using a sterile loop, fresh MTB colonies were transferred to tubes containing glass beads in normal saline. Tubes were vortexed for 60 s then were allowed to stand without agitation for 15 min to allow the beads to settle. Each cell suspension without beads was transferred to a new tube then the turbidity of each suspension was adjusted to 0.5 McFarland standard turbidity using 7H9 broth supplemented with OADC. For MGIT, each suspension was diluted in normal saline by 10^2^-fold (suspension A) and 10^4^-fold (suspension B). Next, 0.5 mL of suspension B was transferred to a drug-free tube for use as the control and 0.5 mL of suspension A was inoculated into a series tubes containing the drug at concentrations ranging from 0.031 to 16 mg/L. Thereafter, all culture tubes were placed in a Bactec MGIT 960 automated system that recorded growth index values automatically. Results were interpreted as follows: when the growth unit (GU) value of the drug-free tube was >400, the isolate was graded as resistant if the GU value of the drug-containing tube was ≥100, otherwise the isolate was graded as susceptible (8). The MIC of each isolate was defined as the lowest drug concentration that was scored as susceptible according to the above-mentioned definition.

For MABA, each suspension was diluted 10-fold with 7H9 medium supplemented with OADC. Then 100 μL of the diluted suspension was added into each well of a microtiter plate containing 100 μl of 2-fold serial dilutions of drugs, such that final drug concentrations in wells ranged between 0.031 mg/L and 32 mg/L. Plates were then covered with self-adhesive membranes and incubated at 37°C in an atmosphere of 5% CO_2_ for 7 days. After 7 days, 70 μL of freshly prepared alamarBlue solution was added to each well, then the plates were re-incubated for an additional 24 h at 37°C. After incubation, wells were checked for a color change from blue to pink that indicated bacterial growth, whereby the MIC was defined as the lowest concentration of drug that prevented a color change (4). Each isolate was tested in triplicate using the same inoculum. The quality of each batch of experiments was evaluated based on whether the MIC of the reference MTB strain H37Rv (ATCC27294), which was tested in parallel with samples, was within the expected range of 0.031 to 0.25 mg/L. Epidemiological cut-off (ECOFF) was defined as the highest MIC value of the wild-type distribution, whereas the critical concentration (breakpoint) was defined as the MIC value that was used to distinguish susceptible from resistant MTB isolates(25).

### Time to detection.

The Bactec MGIT 960 system was used to assess growth rates of RIF-resistant MTB isolates as previously described (8). Briefly, each bacterial suspension was adjusted so that it was equivalent to a McFarland 1.0 turbidity standard as per the description above. Next, 0.5 mL of each 10^3^-fold-diluted suspension was inoculated into an OADC-containing MGIT tube then tubes were incubated at 37°C in the Bactec MGIT 960 system. Time to detection (TTD) was calculated as the interval between the time of inoculation of the suspension into the MGIT tube and the time when the instrument recorded positive growth.

### Statistical analysis.

We performed a paired chi-square test to compare RIF resistance-detection accuracies of MGIT and MABA. In addition, for different isolates we conducted comparisons of TTDs stratified to different MICs using the Student’s *t* test and assessed the linear correlation between MICs and TTD values. We then applied kernel density estimation (KDE), a non-parametric statistical method for estimating the probability distribution of MTB isolates with different MICs (26). Statistical analysis of results was conducted using SPSS 20.0 (IBM Corp., Armonk, NY, USA). Differences with *P values* of ≤0.05 were considered statistically significant.
